# Peer support needs and engagement among people with multiple sclerosis: Associations with social support, loneliness and wellbeing: A cross-sectional study

**DOI:** 10.1177/13591053251392873

**Published:** 2026-01-15

**Authors:** Joan Alaboson, Laura Coffey, Rebecca Maguire

**Affiliations:** 1 Maynooth University, Kildare, Ireland

**Keywords:** peer support, loneliness, wellbeing, social support, multiple sclerosis

## Abstract

Peer support may improve wellbeing in people with multiple sclerosis (PwMS). This study examined associations between peer support, social support, loneliness and wellbeing in PwMS, along with sociodemographic and health predictors of peer support need and engagement in online and in-person contexts. A cross-sectional survey, co-designed with public and patient involvement, was deployed among 218 PwMS in Ireland, with regression analyses used to explore predictors of needs for, and engagement with, peer support. Increasing disability levels and fewer years with MS were associated with higher peer support need. Wellbeing was significantly yet weakly positively correlated with in-person peer support engagement (*r*_s_ = 0.1967, *p* = 0.005). However, only loneliness and social support were significant predictors of wellbeing, accounting for approximately 40% of the variance. Overall, PwMS need peer support, yet reported low engagement with peers. While no clear associations with wellbeing were established, findings suggest that peer support may improve psychosocial experiences in MS.

## Introduction

Multiple sclerosis (MS) is a chronic neurological illness with a variable course, where symptoms may remit and relapse alone, or progressively worsen at any stage (Moreno-Torres et al., 2019). MS onset is typically between 20 and 40 years of age, with a 2:1 female-to-male prevalence ratio (Moreno-Torres et al., 2019). Given its presenting course, MS is classified into relapsing-remitting MS, or progressive MS, where it is secondary progressive if progression follows an initial relapsing remitting period, or primary progressive if symptoms worsen without remit. People with multiple sclerosis (PwMS) consequently experience variable symptoms, with common problems including difficulties with sensory perception, bladder and sexual health, sight, mobility, fatigue, and brain fog, among others (Moreno-Torres et al., 2019).

PwMS may experience poorer psychological outcomes compared to the wider population (Fisher et al., 2020; Solaro et al., 2018). This includes an increased risk of anxiety and depression (Jones et al., 2012), with studies estimating that at least one in five PwMS experience these states (Boeschoten et al., 2017; Marrie et al., 2015). PwMS may also experience a lower quality of life (QoL) linked to concurrent disabilities, fatigue and other manifestations of MS (Gil-González et al., 2020), which may in turn lead to lower psychological wellbeing (Strober, 2018). They additionally may experience low social support, and high levels of isolation and loneliness (Latinsky-Ortiz and Strober, 2022).

In dealing with the impact of MS, PwMS may require care from a range of healthcare professionals, however barriers to accessing such care may be encountered. These barriers may include a lack of appropriate transportation and long commutes to care, which may be provided in buildings with no or limited accommodation to their needs (Chiu et al., 2017; Mayo et al., 2021). Even when PwMS get access to care, a review by Chiu et al. (2017) showed that they reported dissatisfaction and concerns during consultations. For example, concerns over the breadth of MS or disability-related knowledge displayed by healthcare providers were expressed, resulting in some PwMS having substantial unmet support needs.

Aside from a need for appropriate healthcare, psychological interventions may improve the wellbeing of PwMS. Examples of supportive interventions for PwMS include approaches such as mindfulness and cognitive behavioural therapy, which have been shown to improve QoL (Gil-González et al., 2020), depression (Thomas et al., 2006), and psychological wellbeing (Graziano et al., 2014). However, psychological interventions may not always be adequate or accessible to all PwMS. There is a need, therefore, for the development of further interventions to better meet the needs of this cohort. Peer support interventions may be one way to achieve this.

Peer support refers to the unique support offered by peers that is underpinned by understanding or experiencing a shared health condition or life situation (Gerritzen et al., 2022). This includes providing information, practical advice or emotional support, delivered face-to-face or, increasingly, through internet-mediated technologies including websites, social media and teleconferencing media, amongst others (Boyt et al., 2022; Dunn et al., 2003). This support may occur one-to-one or in groups, facilitated by therapists, other health professionals, or PwMS (Dunn et al., 2003; Hossain et al., 2021). Often, these opportunities are facilitated by MS societies and advocacy groups, such as MS Ireland (MSI), a charitable organisation involved in providing a wide range of supports for people affected by MS in Ireland (Maguire et al., 2023).

A growing body of research shows that peer support is potentially effective in improving psychological outcomes in MS (Ng et al., 2013), whether led by health workers or peers themselves, where research suggests more positive effects (Azizi et al., 2020). This includes reducing feelings of loneliness (Leavitt et al., 2020). As such, peer support may help to meet some needs of PwMS and palliate barriers to accessing care while also contributing towards their wellbeing.

Despite its potential benefits, some research among PwMS highlights how a lack of flexibility on meeting times without inclusion of online options can dissuade PwMS from engaging in peer support (McCabe et al., 2015). There is a need to develop a greater understanding of the needs and experiences of PwMS in relation to peer support, as well as how their experiences of peer support are associated with psychological and social outcomes. Understanding these issues could help to guide the development of interventions in this area.

Further justification for peer support development also lies in its connexion to social support. Notable evidence has established a positive relationship between social support and experiences of wellbeing, occurring either from the grounding that social integration may provide generally, or as a reflection of the need-meeting capacity of one’s social circle to mitigate stress (Cohen and Wills, 1985). The latter, involving the functional ability of social contacts to address MS needs, might relate to peers and their unique perspective and shared experience (Mead et al., 2001), and highlight their relevance where this ‘buffer’ is missing.

Given the relationship between loneliness, reduced social support, and poorer wellbeing in PwMS (Latinsky-Ortiz and Strober, 2022), we aimed to explore the relationships between these variables and peer support need and engagement. The primary objectives were to assess (1) need for and engagement in both online and in-person peer support among PwMS in Ireland, (2) socio-demographic and MS-related health predictors of peer support need and engagement, and (3) associations between social support, peer support engagement and psychological wellbeing among PwMS. A secondary objective of this study was to (4) assess the role of peer support engagement in predicting wellbeing, after controlling for socio-demographic, health, and psychosocial factors. To our knowledge this is the first study of its kind to assess the associations, including predictive relationships, between these variables and peer support need and engagement in PwMS. Based on available evidence, we expected that peer support engagement would have positive associations with lower loneliness, and improved wellbeing and social support.

## Methodology

### Design and setting

A cross-sectional mixed methods survey was employed, with reporting adhering to the Strengthening the Reporting of Observational Studies in Epidemiology (STROBE) cross-sectional reporting guidelines (von Elm et al., 2007). A public and patient involvement (PPI) panel was constituted to co-design the study. The PPI panel consisted of seven PwMS (three males, four females, diagnosed 3–44 years ago), who responded to an online invitation to help shape the research project. This invitation was distributed on the PPI Ignite Network (a consortium of Irish universities and stakeholders promoting PPI), and MSI websites and social media platforms. The PPI panel prioritised this study from a selection of potential areas of focus and refined the survey’s questions in November 2023.

The study received ethical approval from the Maynooth University Social Research Ethics Sub-Committee (Ethics Review ID: 37823). Data was collected as part of a larger study that also collected information from informal caregivers of PwMS. Participation in the survey was anonymous and confidential, and followed respondents’ provision of informed consent.

### Recruitment and data collection

Recruitment was conducted between February and March 2024, with purposive and snowball strategies employed. The survey invitation and information links were distributed through MS Ireland, who shared them on their social media platforms, and through their mailing lists and community workers, to increase participation of people who might missed the invitation online. Potential respondents received information about the study, as well as a link to Qualtrics (2020) where interested participants could provide consent and complete the survey electronically. To be eligible to participate, respondents had to be consenting people with a diagnosis of MS, aged 18 years and above, and resident in the Republic of Ireland. Additionally, because of the measures utilised, participants had to be fluent in English. Respondents not meeting these criteria were excluded from participation. Participants received no remuneration for their involvement with this study.

### Measures

The survey consisted of five sections which collected information on (1) socio-demographic and health characteristics, (2) experiences of loneliness, social support and wellbeing, (3) engagement with in-person and online peer support, (4) need, benefits, barriers, and facilitators of peer support engagement, and (5) peer support preferences and confidence using digital devices.

A combination of validated measures and researcher-developed questions was used to collect quantitative data, while open-text questions were used to collect qualitative data. See Supplemental Table S1 for full details on the survey.

*Socio-demographic background and health situation*: Participants were first asked questions about their socio-demographic details. These included: age, gender, ethnicity, residence, living situation, employment status, relationship status, and ease of making ends meet (*a measure on a six-point scale where participants reported the extent to which they could make ends meet, ranging from ‘with great difficulty’ to ‘very easily’*).

Next, participants were asked questions relating to their health. This included information about the duration of living with MS, and MS type. The Patient Determined Disease Steps (PDDS) scale measured participants’ mobility and use of mobility supports or assistance, with responses ranging from ‘normal’ to ‘bedridden’ corresponding to a score of 1–9, respectively (Learmonth et al., 2013). Participants were also asked if they required help or support with their daily activities.

*Social support*: Participant experiences of social support were explored using the three-item Oslo Social Support Scale (OSLO-3: Bøen et al., 2012), which includes questions on close contacts, perceived concern of contacts, and access to support from neighbours. Total scores ranged from 3 to 14 and were classified into poor, moderate and strong, with scores of 3–8, 9–11, and 12–14, respectively. The OSLO-3, used in population-based studies, has a low but acceptable level of internal consistency (Cronbach’s α = 0.640; Kocalevent et al., 2018). It was selected for its brevity, with a 0.63 Cronbach’s α noted in our sample.

*Loneliness*: The UCLA Loneliness Scale (version 3) collected experiences of loneliness (Hughes et al., 2004). Three questions explored experiences of companionship, isolation and social exclusion through Likert responses ‘hardly ever’ to ‘often’ and total scores ranged between 3 and 9, with higher scores suggestive of greater self-reported loneliness. Total scores were further classified into low (3–4), moderate (5–6), and high (7–9) loneliness, respectively. The UCLA-3 has good internal consistency and reliability reported in population-based studies (Cronbach’s α = 0.72 (Hughes et al., 2004) and ranging from 0.89 to 0.94 (Russell, 1996)). The UCLA-3 has a unidimensional structure and has been used to measure loneliness among PwMS (Balto et al., 2019; Kasikci and Dayapoglu, 2021), with a 0.85 Cronbach’s α noted in our sample.

*Wellbeing*: The five-item World Health Organisation Wellbeing Index (WHO-5: (Topp et al., 2015)) collected experiences of wellbeing in the preceding 2-week period. Likert responses ranged from ‘at no time’ to ‘all the time’, equivalent to a score of 0–5, respectively. Total scores ranged from 0 to 25, and were standardised to 0–100, with higher scores suggesting better self-reported wellbeing levels. Participants were also screened for depression risk, based on existing literature that suggests a cut-off score of 50, with lower scores suggesting some risk and scores below 28 suggestive of risk of major depressive episodes (Löwe et al., 2004; Topp et al., 2015). Studies show the WHO-5 has a unidimensional structure with a good level of internal consistency ranging from 0.81 to 0.85 in population-based studies (Fung et al., 2022), and 0.923 in mental health settings (Lara-Cabrera et al., 2020). Cronbach’s α = 0.89 was found in our sample.

*Peer support*: A definition of peer support, based on the work of Gerritzen et al. (2022) and Dunn et al. (2003), was provided to contextualise questions about PwMS’ need for and experiences of peer support delivered both face-to-face and online and standardise recall or interpretation of relevant supports. To identify peer support need, participants indicated either that they do not have a peer support need, a need that is met, or an unmet need for peer support. Their frequency of engagement in online and in-person peer support was assessed in questions with a five-point Likert scale (‘*Never’ [0] to ‘Nearly Always*’ [4]) responses.

Other questions exploring participants’ perceived usefulness, benefits and disadvantages of peer support were explored through researcher-devised Likert and closed-ended questions. Participants also shared their preferences (including delivery mode, group size and composition) for peer support participation face-to-face or online. Open-text questions collected examples of peer support opportunities that participants had engaged in.

### Data analysis

A mixed-methods approach was employed to analyse the survey data. This paper addresses the previously outlined objectives. Descriptive statistics were calculated for all quantitative variables. Means and standard deviations were calculated for continuous variables, while frequencies and percentages were calculated for categorical variables. To meet objective 1, descriptive statistics assessed the level of need for and engagement in both online and in-person peer support.

For the purpose of further analysis, peer support need and engagement were treated as continuous variables and binary variables, where ‘0’ was representative of responses of no peer support need and ‘1’ of any need (either met or unmet). Also, responses of ‘*never*’ having engaged in either in person or online peer engagement were represented with ‘0’, and any level of engagement (‘*rarely*’- ‘*nearly always*’) represented with ‘1’.

Logistic regression analyses were used to achieve objective 2, exploring predictors of peer support need and levels of engagement in peer support opportunities. Peer support need and peer support engagement were the dependent variables with socio-demographic and health-related factors *((1) age, (2) gender, (3) ease of meeting needs (ease, difficulty), (4) employment status, (5) relationship status (in a relationship, others), (6) residence, (7) living situation (living alone or with others), (8) years lived with MS, (9), and MS type (10) disability*) as predictors. Ethnicity was excluded from all analyses, since 99.1% of participants identified as Caucasian/White.

Pearson correlations were used to assess associations between peer support need and engagement, social support, loneliness and psychological wellbeing (objective 3). Correlation analysis showed no multicollinearity; the assumptions of linearity and homoscedasticity were also met.

Finally, to meet the secondary study objective assessing the role of peer support engagement in predicting wellbeing, after controlling for socio-demographic, health, and psychosocial factors, a hierarchical multiple regression model was developed. It assessed relationships between WHO-5 scores (psychological wellbeing) and five blocks of predictors: (1) socio-demographic factors, namely age, gender (*male, female, third gender*), ease of meeting needs (*ease, difficulty*), employment status (*employed full-/part-time, unemployed*), relationship status (*polyamorous/in any relationship, other*), residence (*urban, rural*), and living situation (*living alone, other*), (2) health factors, namely years with MS, MS type (*progressive MS, other*), and PDDS score/disability, (3) OSLO-3 scores (social support), (4) in-person and online peer support engagement frequencies (*never, other*), and (5) UCLA-3 scores (loneliness).

Responses to categorical variables were converted into binary responses described above, and participants with incomplete or missing data across the variables were excluded from the analysis. RStudio Software (Team R, 2024) was used in the analysis. Using G*Power Software (Faul et al., 2007), a sample size for multiple regression, to yield a power of at least 0.80 with a significance level of 0.05, detecting a medium effect size of 0.15 was calculated to be 123 PwMS (11 predictors) for the primary analyses. To achieve the secondary objective, using G*Power Software, a sample size of 194 testing 14 predictors was calculated to yield a power of 0.95, with a 0.05 significance level, detecting a medium effect size of 0.15.

## Results

### Descriptive statistics

A total of 218 participants responded to the survey. Table 1 provides descriptive statistics summaries of the study variables.

**Table 1.** Socio-demographic and health characteristics.

Variable	Category	*N* (%), *N* = 218	Valid %	Missing (%)
Gender	Male	50 (23)	23	1 (0.5)
	Female	166 (76)	76.5	
	Third gender/non-binary	1 (0.5)	0.5	
Ethnicity	Caucasian/White	214 (98.2)	99.1	2 (0.9)
	Other (*Catholic, Irish*)	2 (0.9)	0.9	
Residence	Rural	103 (47.2)	47.9	3 (1.4)
	Urban	112 (51.4)	52.1	
Living situation: Do you live alone?	No, I live with family	167 (76.6)	77.3	2 (0.9)
	No, I live with flat/housemates	11 (5.1)	5.1	
	Yes, I live alone	38 (17.4)	17.6	
Relationship status	Married/cohabiting	144 (66.1)	66.7	2 (0.9)
	In a relationship but not cohabiting	10 (4.6)	4.6	
	Other	62 (28.6)	28.7	
Employment status	Employed full-time	57 (26.1)	26.3	1 (0.5)
	Employed part-time	34 (15.6)	15.7	
	Homemaker	31 (14.2)	14.3	
	Retired	59 (27.1)	27.2	
	Student	3 (1.4)	1.4	
	Unemployed	33 (15.1)	15.2	
Ease of making ends meet (Income)	Very easily	16 (7.3)	7.4	2 (0.9)
	Easily	45 (20.6)	20.8	
	Fairly easily	79 (36.2)	36.6	
	With some difficulty	49 (22.5)	22.7	
	With difficulty	17 (7.8)	7.9	
	With great difficulty	10 (4.6)	4.8	
MS type	Relapsing remitting MS	141 (64.7)	65.6	3 (1.4)
	Secondary progressive MS	34 (15.6)	15.8	
	Primary progressive MS	26 (11.9)	12.1	
	Benign MS	1 (0.5)	0.45	
	Unsure	11 (5.1)	5.1	
	Unclear answer	2 (0.9)	0.9	
Self-reported care or support requirement	Yes	103 (47.3)	48.6	6 (2.8)
	No	109 (50)	51.4	
Peer support need	Have no need for peer support	72 (33)	39.1	34 (15.6)
	Have a peer support need currently met	63 (28.9)	34.2	
	Have an unmet peer support need	49 (22.5)	26.6	
In-person peer support engagement	Never	77 (35.3)	37	10 (4.6)
	Rarely	66 (30.3)	31.7	
	Sometimes	43 (19.7)	20.7	
	Quite frequently	17 (7.8)	8.2	
	Nearly always	5 (2.3)	2.4	
Online peer support engagement	Never	61 (28)	30.3	17 (7.8)
	Rarely	42 (19.3)	20.9	
	Sometimes	64 (29.4)	31.8	
	Quite frequently	28 (12.8)	13.9	
	Nearly always	6 (2.8)	3	
OSLO-3/Social support (*M* = 9.08; SD = 2.40)	Poor social support	87 (39.9)	41.4	8 (3.7)
	Moderate social support	93 (42.7)	44.3	
	Strong social support	30 (13.8)	14.3	
UCLA-3/Loneliness (*M* = 5.16; SD = 1.74)	Low loneliness	74 (33.9)	35.2	8 (3.7)
	Moderate loneliness	98 (45)	46.7	
	High loneliness	38 (17.4)	18.1	
WHO-5/Depression risk (*M* = 43.2; SD = 23)	Low or no depression risk	73 (33.5)	35.4	12 (5.5)
	Some depression risk	65 (29.8)	31.6	
	High depression risk	68 (31.2)	33	

#### Sociodemographic and health characteristics

The sample consisted predominantly of females (76.5%) and Caucasians (99.1%), with a mean age of 49.31 years (SD = 12.25), ranging between 22 and 79 years. The majority were living with RRMS (65.6%), aligning with MS sub-type prevalence (Ghasemi et al., 2017). Approximately seven out of ten respondents were in a relationship, and lived with family. Participants had a mean of 13.18 years since their MS diagnosis (SD = 9.57), with a range of 0–44 years. Although PDDS scores ranged between one (normal) and nine (bedridden), participants had a mean score of 3.89 (SD = 2.54), equivalent to people experiencing a gait disability where assistance might be required during a relapse (Learmonth et al., 2013). Almost equal numbers of participants in the sample resided in urban (52.1%) versus rural (47.9%) areas, and 48.6% reported a need for care or support in their daily activities. Just under half of participants (42%) were in some form of employment, with approximately 35% reporting some level of difficulty in making ends meet.

#### Social support, loneliness and wellbeing

The mean OSLO-3 score was 9.08 (SD = 2.4), with 41.4% of participants reporting poor social support. The mean UCLA-3 scores were 5.16 (SD = 1.74), ranging between 3 and 9, with 35.2% reporting low levels of loneliness. The mean standardised WHO-5 score was 43.2 (SD = 22.99), indicative of lower wellbeing in the sample compared to the general population. A significant number of participants had some (31.6%) depression risk or major (33%) depression risk.

#### Peer support need and engagement

Approximately a third (39.1%) of participants expressed no need for peer support. However, 60.8% expressed a need for peer support, about half (26.6%) of whom had these needs unmet. Participants were slightly more likely to have engaged in online (69.6%) than in-person (63%) peer support, with 30.3% never having engaged online compared to 37% reporting no in-person peer support engagement. Engagement was infrequent, however; only 31.3% of participants availed of in-person peer support sometimes or more frequently (see Table 1).

### Logistic regression analysis

#### Peer support need

There was a significant effect (*p* = 0.01) of increasing disability on the odds of peer support need increasing, holding all other predictor variables constant, with an odds ratio of 1.36, 95% confidence interval (CI)(1.03 e00, 1.86). There was also a significant effect (*p* = 0.01) of increasing years lived with MS on the odds of peer support need decreasing, holding all other predictor variables constant, with an odds ratio of 9.09e−01, 95% CI (8.42e−01, 0.97). Other predictors had insignificant effects on the odds of peer support need. Overall, the model had a significant effect (*p* = 0.01) on peer support need (χ^2^ = 24.13), with between 22.2% (Cox and Snell) and 30.6% (Nagelkerke) of variance explained.

#### In-person peer support engagement

There was a significant effect (*p* = 0.01) of increasing disability, holding all other predictor variables constant, on the odds of in-person peer support engagement increasing with an odds ratio of 1.47, 95% CI (1.10, 2.03). Other predictors had insignificant effects on the odds of in-person peer support. Overall, the model had an insignificant effect (*p* = 0.22) on in-person peer support (χ^2^ = 14.20), with between 9.5% (Cox and Snell) and 18.6% (Nagelkerke) of variance explained.

#### Online peer support engagement

There was a significant effect (*p* = 0.000) of increasing disability, holding all other predictor variables constant, on the odds of online peer support engagement increasing with an odds ratio of 1.94, 95% CI (1.36, 2.96). There was also a significant effect (*p* = 0.01) of increasing years lived with MS, holding all other predictor variables constant, on the odds of online peer support engagement decreasing with an odds ratio of 8.97e−01, 95% CI (8.16e−01, 0.97). Other predictors had insignificant effects on the odds of online peer support. Overall, the model had a significant effect (*p* = 0.01) on online peer support (χ^2^ = 24.12), with between 22.2% (Cox and Snell) and 32.2% (Nagelkerke) of variance explained.

### Pearson correlations

Associations between loneliness, social support, peer support and psychological wellbeing.

The was a significant medium positive correlation between engagement in in-person and online peer support (*r*_s_ = 0.489, *p* = 0.000). See Supplemental Table S2 for details.

Psychological wellbeing was significantly yet weakly positively correlated with engagement with in-person peer support (*r*_s_ = 0.1967, *p* = 0.005). It also had an insignificant weak positive correlation with engagement with online peer support. Loneliness had a significant weak negative correlation with frequency of engagement with in-person support (*r*_s_ = −0.207, *p* = 0.003), but did not correlate with any frequency of online peer support engagement (*r*_s_ = 0.05, *p* = 0.46). Social support was weakly positively correlated with engagement in both in-person (*r*_s_ = 0.170, *p* = 0.016) and online (*r*_s_ = 0.181, *p* = 0.011) peer support. It also had a significant medium positive correlation with psychological wellbeing (*r*_s_ = 0.492, *p* = 0.00).

### Hierarchical regression analysis

A hierarchical regression analysis was conducted in five blocks to explore the role of peer support engagement in psychological wellbeing after controlling for known sociodemographic, health and psychosocial associates. Block 1, comprising sociodemographic and Block 2, comprising health-related factors, were not significant, predicting only 7.7% and 9% of the variance in standardised WHO-5 (wellbeing) scores, *F*(8, 132) = 1.384; *p* = 0.203, and *F*(11,129) = 1.164; *p* = 0.318, respectively. Block 3, consisting of OSLO-3 (social support), was significant, predicting 32.9% of the standardised WHO-5 score variance, *F*(12, 128) = 5.23, *p* = 4.117^e−07^. This model also showed a significant improvement from the second model (*R*^2^ change = 0.24, χ^2^ = 50.37, *p* = 8.43^e−11^). Block 4, comprising online and in-person peer support engagement, was not significant; however, it predicted 33.2% of variance (*R*^2^) in WHO-5 scores, *F*(14, 126) = 4.482, *p* = 1.729^e−06^. The final Block comprised UCLA-3 (loneliness) scores, which were significant in predicting 40.7% of the variance (*R*^2^) in WHO-5 scores, *F*(15, 125) = 5.73, *p* = 7.9^e−09^. It showed a significant improvement from the fourth model (*R*^2^ change = 0.07, χ^2^ = 15.79, *p* = 0.0001). See Table 2. The strongest predictors of wellbeing in order of magnitude were social support, *β* = 5.47, *p* = 4.63^e−10^, then loneliness *β* = −4.86, *p* = 0.0001.

**Table 2.** Hierarchical regression analysis of WHO-5 (wellbeing) score predictors.

Variables	*β*	*p*	*t*	SE	95% CI
Step 1: Sociodemographic factors					
Age	0.24	0.203	1.28	0.19	−0.13, 0.62
Gender—male	− .03	0.994	−0.01	4.93	−9.78, 9.71
Gender—Binary/third gender	30.79	0.203	1.28	24.05	−16.79, 78.37
Ease of meeting needs—ease	8.11	0.072	1.82	4.47	−0.72, 16.95
Employment status—other	− 7.46	0.109	−1.61	4.62	−16.61, 1.68
Relationship status—other	− 4.51	0.417	−0.81	5.55	−15.49, 6.46
Residence—urban	0.66	0.873	0.16	4.15	−7.55, 8.87
Living situation—other	− 5.84	0.371	−0.90	6.50	−18.71, 7.03
Adjusted *R*^2^ = *0.0215*					
Step 2: Health factors					
Years with MS	− 0.06	0.828	−0.22	0.26	−0.63, 0.50
MS type—progressive MS	−8.51	0.186	−1.33	6.41	−21.19, 4.16
Disability	1.18	0.323	0.99	1.20	−1.18, 3.56
Adjusted *R*^2^ = *0.0127*					
Adjusted *R*^2^ change = 0.013					
Step 3: Social support					
OSLO-3 scores	5.47	4.63^e−10^***	6.75	0.81	3.87, 7.07
Adjusted *R*^2^ = *0.266*					
Adjusted *R*^2^ change = 0.238					
Step 4: Peer support (PS)					
Online peer support engagement—other	−2.71	0.553	−0.59	4.56	−5.19, 11.19
In-person peer support engagement—other	3.00	0.469	0.73	4.14	−11.72, 6.31
Adjusted *R*^2^ = *0.258*					
Adjusted *R*^2^ change = 0.003					
Step 5: Loneliness					
UCLA scores	−4.86	0.0001***	−3.97	1.22	−7.28, −2.44
Adjusted *R*^2^ = *0.336*					
Adjusted *R*^2^ change = 0.075					

Gender—male/female/third gender; Ease of meeting needs—ease/difficulty; Employment status—employed/other; Relationship status—in a relationship/other; Residence—urban/rural; Living situation—living alone/other; MS type—progressive MS/other; Online peer support engagement—never/other frequency; In-person peer support engagement—never/other frequency.

*** *p* = 0.000.

## Discussion

This study has established that many PwMS require peer support, and that a significant proportion of PwMS in Ireland have unmet needs for such support. However, the level at which PwMS engage with peers may not be reflective of this need. Findings also highlight how PwMS may experience challenging psychosocial circumstances that have a relationship with their levels of peer support engagement. We showed that peer support is particularly needed for PwMS at higher levels of disability and those at an earlier stage of their diagnosis. Despite this, our findings suggest that engaging in peer support has a limited role in the wellbeing of PwMS, necessitating further study to explore other potential predictors.

Notably, findings suggest that almost two-thirds of PwMS desire peer support, which is consistent with previous studies among PwMS in Australia (McCabe et al., 2015), and in cancer survivors, where over 70% of participants in a recent cross-sectional survey reported peer support need (Bender et al., 2022). However, while 63% and 70% of our sample engaged in some form of in-person and online peer support respectively, which closely matches the reports of a large cross-sectional study among PwMS aged 45 years and above (Finlayson and Cho, 2011), we found that this engagement dropped dramatically (31.7 and 21% points, for in-person and online peer support respectively), where engagement frequencies of sometimes or more were considered. Evidently, peer support engagement was low.

Some studies have highlighted a need for flexibility in timing and a wider range of peer support opportunities (McCabe et al., 2015). These needs could be met by providing a variety of online peer support options that eliminate transportation and logistical challenges accompanying in-person peer support identified in Gerritzen et al.’s review (2022). We found a predilection for online engagement in our sample, that may be explained by these benefits.

The only factor which significantly and positively predicted peer support need and engagement was increasing disability. In view of Mead et al.’s (2001) theoretical perspective on peer support, we see that PwMS might seek support from people with similar experiences or perspectives on their changing lives with MS-related disability. This is echoed in other research, Finlayson and Cho (2011), who explored professional-led support group use among PwMS in the United States and found a greater likelihood of attendance in groups with limitations in maximal instrumental activities and daily living. We add to the literature specifically showing that, as PwMS experience limitations in their physical care and independent living or external responsibilities, not only might they require assistance from family and close contacts (Borreani et al., 2014), they also require the same from peers. This finding is notable given that the majority of participants in our sample had RRMS and were largely able to walk without difficulty and did not need assistance, except during a relapse.

Conversely, we found that those who were more recently diagnosed were more likely to report an increased need for support, along with increased online engagement. These findings suggest then that there are potentially two distinct groups in need of peer support: PwMS with higher levels of disability and those at an earlier stage of diagnosis. This is consistent with broader literature in the area of MS, suggesting that these two cohorts are in greatest need of support. Following diagnosis, PwMS experience lower QoL that may be related to experiences of anxiety and symptom intensity (Strober, 2018); but studies have demonstrated better QoL in PwMS with a longer MS duration (Schwartz and Frohner, 2005). Likewise, with higher disability levels, Hakim et al. (2000) highlighted that PwMS reported a shrinkage of social engagements, even with former friends. These findings were more recently echoed in a cross-sectional study amongst PwMS and community workers in Ireland, where both groups highlighted that support was needed around diagnosis, with community workers facilitating the connection between peers (Maguire et al., 2023).

Peers can serve different functions at these critical times. Having a mutual lived understanding of challenges, peers can provide relevant information and emotional support (Gerritzen et al., 2022). They then go on to support peers as MS symptoms progress, supporting information and instructional needs on how to avail of benefits, access equipment or assistive technology, and secure home modifications, or to get on living with progressive MS symptoms (Maguire et al., 2023). Overall, they can contribute to improving the social network of PwMS, and thus potentially offer avenues to exchange social support and reduce loneliness, associations that we also found in our study.

For organisations that seek to facilitate peer support, these findings provide a clear starting point in identifying people who might require support. Even where peer support is not considered, but professional or psychotherapy supports are made available, (Morris-Bankole and Ho, 2023) in their cross-sectional survey showed that 30%–40% of PwMS still engage with group psychotherapy and patient education. These are also deliverable through peer support.

Our study highlighted generally weak to medium relationships between wellbeing, engagement in peer support and other psychosocial circumstances experienced by PwMS. The weak yet positive relationship established between in-person peer support engagement and psychological wellbeing suggests in-person peer support’s potential benefits on mood. Despite this, our findings underscore the need to provide emotional or psychological support to PwMS, with a significant number of participants having some (31.6%) or major (33%) depression risk. Ng et al. (2013) also demonstrate that in-person peer support approaches are beneficial in reducing negative psychological outcomes, including depression among PwMS. In cancer, with a history of more established peer support programmes or approaches, recent systematic reviews have shown that there are minimal (Kiemen et al., 2023) to significant (Zhang et al., 2022) benefits of peer support on depression.

Our study advances insights on the relationship between greater in-person peer support engagement and reduced experiences of loneliness, as well as improvements in self-reports of social support following increases in both in-person and online peer support. This is unsurprising, in view of the fact that people without MS might not understand MS-related topics or issues, and may not be seen to accommodate nor support PwMS the way peers can (Cohen and Wills, 1985; Mead et al., 2001). There is also abundant evidence linking the intensity of MS symptoms such as pain and fatigue to social participation and loneliness (Kratz et al., 2017; Latinsky-Ortiz and Strober, 2022), and of progressive disability to lower social support (Hakim et al., 2000). In other words, PwMS may not be able to keep their other social contacts, with dire consequences. These findings further suggest how peer support could have multidimensional psychological impacts.

Finally, we show that peer support has the potential to increase social support and reduce loneliness, both of which were found to predict wellbeing in this sample, with the final model exploring the added role loneliness plays, accounting for 33.6% of the variance in wellbeing scores. Our exploration of social support using the OSLO-3 focussed largely on the numbers or quantity of available support, and may be in line with Cohen and Willis’s (1985) hypothesis, where wellbeing improves with PwMS finding a stable network where they are grounded and maintain a positive identity (the main effect). Taken together with our other findings involving peer support, a more qualitative assessment of social support and or specific aspects of peer support is required to explore its wider contributions to wellbeing in MS. More robust research is needed to clarify these relationships, as well as other modifiable correlates of PwMS’s wellbeing.

### Study implications and limitations

Future research could try to identify and include other factors that were not included in this model. For example, PwMS perception of their functional deficits as a component of cognitive dysfunction could be added. Ryan et al. (2007) in their cross-sectional study found that PwMS self-reported cognitive functional deficits, social support, and the difference between PwMS and their carers’ perception of their functional deficits (deficit unawareness), predicted 40% of subjective wellbeing variance, with the deficit unawareness contributing 18% to this variance. Further, Kratz et al. (2017) found that fatigue was associated with wellbeing in a multivariate multilevel model examining associations between MS symptoms and wellbeing. Fatigue could equally be added. Although our study focused on psycho-social factors, these broader MS challenges may play an additional role in explaining PwMS’s wellbeing. Finally, there is a need to standardise and validate objective measures of peer support, and explore their relationships to existing social support measures.

Additionally, other research could delineate experiences of engaging with peer support at different stages of the MS trajectory, exploring its links to wellbeing. Overall, our findings signify a potential for peer support to improve psychosocial outcomes. However, further studies must explore its effectiveness, given the limited existing evaluations of peer support and reports that not all PwMS experience improved wellbeing following peer support engagement (Messmer Uccelli et al., 2004). Finally, organisations mediating or organising peer interactions in MS should consider who might be excluded from accessing these supports in-person and design evidenced-based alternatives.

Our study has several strengths. This is the first study exploring the correlates of wellbeing and peer support in PwMS. We highlight significant associations between peer support, loneliness and social support that have direct implications for the design of current support approaches. We also show that PwMS with increasing disability or at earlier periods following diagnosis are more likely to need and engage with peers. This is complementary to studies in cancer, where peer support engagement is seen to occur more frequently within the years following diagnosis compared to later (Campbell et al., 2004; Kiemen et al., 2023).

However, there are limitations to our study that must be considered. We have investigated self-reports of wellbeing, psychosocial and peer support factors, subject to participants providing responses they consider acceptable. Our findings may thus not accurately represent the experiences of this population. Moreover, there was no verification of MS diagnosis in participants, given it that was deployed online. Further, the questions about sleep and vigour capturing self-reported wellbeing by the WHO-5 (Topp et al., 2015) might limit overall wellbeing assessments, given that fatigue and mobility are common MS symptoms (Gil-González et al., 2020). Although using the OSLO-3 was useful for its brevity, it also had a limited ability to capture all aspects of social support (Cohen and Wills, 1985). The lack of a validated measure of peer support limits its objective measurement, although it is offset by the PPI co-design of this exploratory survey. Although participants’ gender characteristics followed general MS population expectations, there was limited male participation, and only one participant identified as non-binary gender. We also note the wide ranges between the age of participants and their reported time since diagnosis, which might have impacted on their needs and experiences. Finally, by deploying an online survey, this study potentially missed a cohort of PwMS unfamiliar with or without access to the internet. Consequently, further studies are needed to validate this study’s findings among PwMS, and in the broader MS population.

## Conclusion

Peer support is potentially beneficial in supporting PwMS. This study found that PwMS with increasing disability and fewer years with MS have higher peer support needs and engagement. This study’s findings point to important factors to consider in the design of peer support approaches, which are relevant for MS advocacy and support groups. While peer support did not predict wellbeing overall, psychosocial factors that had significant relationships with peer support did. Further robust methodologies can clarify causality between these covariates and test the impact of peer support on wellbeing among PwMS.

## Supplemental Material

sj-docx-1-hpq-10.1177_13591053251392873 – Supplemental material for Peer support needs and engagement among people with multiple sclerosis: Associations with social support, loneliness and wellbeing: A cross-sectional study Supplemental material, sj-docx-1-hpq-10.1177_13591053251392873 for Peer support needs and engagement among people with multiple sclerosis: Associations with social support, loneliness and wellbeing: A cross-sectional study by Joan Alaboson, Laura Coffey and Rebecca Maguire in Journal of Health Psychology

sj-docx-2-hpq-10.1177_13591053251392873 – Supplemental material for Peer support needs and engagement among people with multiple sclerosis: Associations with social support, loneliness and wellbeing: A cross-sectional study Supplemental material, sj-docx-2-hpq-10.1177_13591053251392873 for Peer support needs and engagement among people with multiple sclerosis: Associations with social support, loneliness and wellbeing: A cross-sectional study by Joan Alaboson, Laura Coffey and Rebecca Maguire in Journal of Health Psychology
