# Supplementary material for: Peer support needs and engagement among people with multiple sclerosis: Associations with social support, loneliness and wellbeing: A cross-sectional study
Source: J Health Psychol. 2026 Jan 15;31(9):3777–91. doi: 10.1177/13591053251392873 (PMC13365319; doi:10.1177/13591053251392873)
Supplement: sj-docx-2-hpq-10.1177_13591053251392873 – Supplemental material for Peer support needs and engagement among people with multiple sclerosis: Associations with social support, loneliness and wellbeing: A cross-sectional study [file sj-docx-2-hpq-10.1177_13591053251392873.docx]

**Supplementary Materials**

**(Supplementary material) Table S1** Survey questions

Start of Block: Default Question Block

Q1 What best describes you

I live with multiple sclerosis (MS) (1)

I am a caregiver of someone who lives with multiple sclerosis (2)

I neither live with nor care for someone who lives with multiple sclerosis (3)

End of Block: Default Question Block

Start of Block: Demographic

Q2 Part 1: Demographics

Q3 What gender do you identify with?

Male (1)

Female (2)

Non-binary/ third gender (3)

Prefer not to say (4)

Other (5)

Q4 What age are you?

________________________________________________________________

Q5 What best describes your ethnicity?

Asian (1)

Black/ African/ Carribean (2)

Caucasian/ White (3)

Mixed (4)

Other (5)

Display This Question:

If Q5 = Other

Q6 Please specify your ethnicity

________________________________________________________________

Q7 How would you describe your usual residence?

Urban (1)

Rural (2)

Q8 Do you live alone?

No (1)

Yes (2)

Q9 What best describes your marital or relationship status?

Married/ cohabiting (1)

In a relationship but not cohabiting (2)

Widowed (3)

Single (4)

Other (5)

Display This Question:

If Q9 = Other

Q10 Please specify your marital or relationship status

________________________________________________________________

Q11 What best describes your employment status?

Employed full time (1)

Employed part time (2)

Unemployed (3)

Retired (4)

Student (5)

Homemaker (6)

Q12 Considering your household's total monthly income, how easily can you make ends meet?

Very easily (1)

Easily (2)

Fairly easily (3)

With some difficulty (4)

With difficulty (5)

With great difficulty (6)

Display This Question:

If Q1 = I am a caregiver of someone who lives with multiple sclerosis

Q43 A caregivers in our context includes anyone [partners, family or friends] providing any form of care, help, and or support to someone living with MS. This could include a range of activities such as help with daily personal tasks such as dressing up, providing support while walking, dropping off to appointments, or minding their general needs or wellbeing. Caregivers, in this context, are not employed to formally provide care for the person living with MS and or may not be health and care workers.

Display This Question:

If Q1 = I am a caregiver of someone who lives with multiple sclerosis

Q13 Do you provide regular unpaid personal help or support to a partner, family member, neighbor or friend with MS?

No (1)

Yes (2)

End of Block: Demographic

Start of Block: Health and care requirements

Q14 Part 2: Health and care requirements

Display This Question:

If Q1 = I am a caregiver of someone who lives with multiple sclerosis

Q15 Who is the person with MS that you provide care for

Parent (1)

Partner or Spouse (2)

Offspring (3)

Sibling (4)

Friend (5)

Other (6)

Display This Question:

If Q15 = Other

Q16 Please specify the person with MS you provide care for

________________________________________________________________

Display This Question:

If Q1 = I am a caregiver of someone who lives with multiple sclerosis

Q17 For how many hours per week do you provide care? Write in the space the hours. [For example; care provided on a 24-hour bases, 7 days a week equates to 168 hours]

________________________________________________________________

Display This Question:

If Q1 = I am a caregiver of someone who lives with multiple sclerosis

Q18 Please read the following questions and choose the option that best describes your situation in response to the question.

Display This Question:

If Q1 = I am a caregiver of someone who lives with multiple sclerosis

Q19 I. Do you feel that because of your involvement with your relative/ person requiring care that you do not have enough time for yourself?

Never (1)

Rarely (2)

Sometimes (3)

Quite frequently (4)

Nearly always (5)

Display This Question:

If Q1 = I am a caregiver of someone who lives with multiple sclerosis

Q20 II. Do you feel that because of your involvement with your relative/ person requiring care that you feel stressed between trying to give to your relative/ person requirng care as well as to other family responsibilities, job, etc

Never (1)

Rarely (2)

Sometimes (3)

Quite frequently (4)

Nearly always (5)

Display This Question:

If Q1 = I am a caregiver of someone who lives with multiple sclerosis

Q21 III. Do you feel that because of your involvement with your relative/ person requiring care that you feel strained in your interaction with your relative/ person requiring care

Never (1)

Rarely (2)

Sometimes (3)

Quite frequently (4)

Nearly always (5)

Display This Question:

If Q1 = I am a caregiver of someone who lives with multiple sclerosis

Q22 IV. Do you feel uncertain about what to do about your relative/ person requiring care?

Never (1)

Rarely (2)

Sometimes (3)

Quite frequently (4)

Nearly always (5)

Display This Question:

If Q1 = I live with multiple sclerosis (MS)

Q23 How many years ago were you diagnosed with MS?

________________________________________________________________

Display This Question:

If Q1 = I live with multiple sclerosis (MS)

Q24 What type of MS do you live with?

Relapsing remitting MS (1)

Secondary progressive MS (2)

Primary progressive MS (3)

Unsure (4)

Other (5)

Display This Question:

If Q24 = Other

Q25 Please specify MS type

________________________________________________________________

Display This Question:

If Q1 = I live with multiple sclerosis (MS)

Q26 Please read the choices listed below and choose the one that best describes your own situation.

This scale focuses mainly on how well you walk. You might not find a description that reflects your condition exactly, but please mark the one category that describes your situation the closest.

NORMAL: I may have some mild symptoms, mostly sensory due to MS but they do not limit my activity. If I do have an attack, I return to normal when the attack has passed. (1)

MILD DISABILITY: I have some noticeable symptoms from my MS but they are minor and have only a small effect on my lifestyle (2)

MODERATE DISABILITY: I don't have any limitations in my walking ability. However, I do have significant problems due to MS that limit daily activities in other ways (3)

GAIT DISABILITY: MS does interfere with my activities, especially my walking. I can work a full day but athletic or physically demanding activities are more difficult than they used to be. I usually don't need a cane or other assistance to walk, but I might need some assistance during an attack (4)

EARLY CANE; I use a cane or a single crutch or some other form of support ( such as touching a wall or leaning on someone's arm) for walking all the time or part of the time, especially when walking outside. I think I can walk 25 feet in 20 seconds without a cane or crutch. I always need some assistance ( cane or crutch) if I want to walk as far as 3 blocks. (5)

LATE CANE: To be able to walk 25 feet, I have to have a cane, crutch or someone to hold onto. I can get around the house or other buildings by holding onto furniture or touching the walls for support. I may use a scooter or wheelchair if I want to go greater distances (6)

BILATERAL SUPPORT; To be able to walk as far as 25 feet I must have 2 canes or crutches or a walker. I may use a scooter or wheelchair for longer distances. (7)

WHEELCHAIR/ SCOOTER: My main form of mobility is a wheelchair. I may be able to stand and/ or take one or two steps, but I can't walk 25 feet, even with crutches or a walker. (8)

BEDRIDDEN: Unable to sit in a wheelchair for more than one hour (9)

Display This Question:

If Q1 = I live with multiple sclerosis (MS)

Q27 Do you ever require help, care or support from others to carry out your daily activities?

No (1)

Yes (2)

End of Block: Health and care requirements

Start of Block: Block 3

Q28 Part 3: Your experiences of loneliness/ social support/ wellbeing

Q37 The next questions are about how you feel about different aspects of your life. For each one, please select how often you feel that way – UCLA-3 questions.

Q33 – Q35 OSLO-3 questions

Q36 WHO-5 questions

End of Block: Block 3

Start of Block: Block 4

Q38 Part 4: Your engagement with peer support

Display This Question:

If Q1 = I live with multiple sclerosis (MS)

Q39 Peer support is an exchange of support between people who have a similar health condition or life experience.  Peer support can take place in person (e.g. meet-ups arranged by MS Ireland) or online (e.g. through social media, like MS Facebook groups, or through other online programmes). Given this definition:

Display This Question:

If Q1 = I am a caregiver of someone who lives with multiple sclerosis

Q40 Peer support is an exchange of support between people who have a similar life experience (e.g. support from other caregivers of someone living with MS). Peer support can take place in person (e.g. meet-ups arranged by MS Ireland) or online (e.g. through social media, like MS Facebook groups, or through other online programmes). Given this definition:

Display This Question:

If Q1 = I am a caregiver of someone who lives with multiple sclerosis

Q41 How often do you avail of in-person support from other caregivers of people with MS?

Never (1)

Rarely (2)

Sometimes (3)

Quite frequently (4)

Nearly always (5)

Skip To: End of Block If Q41 = Never

Display This Question:

If Q1 = I live with multiple sclerosis (MS)

Q42 How often do you avail of in-person support from other people with MS?

Never (1)

Rarely (2)

Sometimes (3)

Quite frequently (4)

Nearly always (5)

Skip To: End of Block If Q42 = Never

Q44 What forms of in-person supports have you engaged with?

Click to write Choice 1 (1) __________________________________________________

Click to write Choice 2 (2) __________________________________________________

Click to write Choice 3 (3) __________________________________________________

Q45 Please rate your agreement with the statement:  'I have found in-person supports to be beneficial'.

Strongly agree (1)

Agree (2)

Neither agree nor disagree (3)

Disagree (4)

Strongly disagree (5)

End of Block: Block 4

Start of Block: Block 6

Display This Question:

If Q41 = Never

Q48 If never, why not?

________________________________________________________________

Display This Question:

If Q42 = Never

Q49 If never, why not?

________________________________________________________________

End of Block: Block 6

Start of Block: Block 5

Display This Question:

If Q1 = I live with multiple sclerosis (MS)

Q46  How often do you avail of online support from other people with MS? Online support could include websites, social media (e.g. Facebook groups), or online events hosted on platforms like Zoom or Skype.

Never (1)

Rarely (2)

Sometimes (3)

Quite frequently (4)

Nearly always (5)

Skip To: End of Block If Q46 = Never

Display This Question:

If Q1 = I am a caregiver of someone who lives with multiple sclerosis

Q47  How often do you avail of online support from other caregivers of people with MS? Online support could include websites, social media (e.g. Facebook groups), or online events hosted on platforms like Zoom or Skype.

Never (1)

Rarely (2)

Sometimes (3)

Quite frequently (4)

Nearly always (5)

Skip To: End of Block If Q47 = Never

Q50 What forms of online supports have you engaged with?

Click to write Choice 1 (1) __________________________________________________

Click to write Choice 2 (2) __________________________________________________

Click to write Choice 3 (3) __________________________________________________

Q51 Please rate your agreement with the statement:  'I have found online supports to be beneficial'.

Strongly agree (1)

Agree (2)

Neither agree nor disagree (3)

Disagree (4)

Strongly disagree (5)

Q52 What are the main benefits, if any, of engaging in peer support?

________________________________________________________________

Q53 What are the main disadvantages, if any, of engaging in peer support?

________________________________________________________________

End of Block: Block 5

Start of Block: Block 7

Display This Question:

If Q46 = Never

Q54 If never, why not?

________________________________________________________________

Display This Question:

If Q47 = Never

Q55 If never, why not?

________________________________________________________________

End of Block: Block 7

Start of Block: Block 8

Q57 Which of the following best applies to you? ( Check the one that applies)

Display This Choice:

If Q1 = I live with multiple sclerosis (MS)

I do not have a need for peer support from other people with MS (1)

Display This Choice:

If Q1 = I am a caregiver of someone who lives with multiple sclerosis

I do not have a need for peer support from other caregivers of people living with MS (2)

Display This Choice:

If Q1 = I live with multiple sclerosis (MS)

I need peer support from other people with MS, but these needs are currently met (3)

Display This Choice:

If Q1 = I am a caregiver of someone who lives with multiple sclerosis

I need peer support from other caregivers of people with MS, but these needs are currently met (4)

Display This Choice:

If Q1 = I live with multiple sclerosis (MS)

I need peer support from other people with MS, but these needs are unmet or not fully met (5)

Display This Choice:

If Q1 = I am a caregiver of someone who lives with multiple sclerosis

I need peer support from other caregivers of people with MS, but these needs are unmet or not fully met (6)

Q56 If applicable what prevents you from participating or deciding to participate with peer support?

________________________________________________________________

Display This Question:

If Q1 = I am a caregiver of someone who lives with multiple sclerosis

Q59 What barriers does your care recipient currently experience participating or deciding to participate with peer support?

Click to write Choice 1 (1) __________________________________________________

Click to write Choice 2 (2) __________________________________________________

Click to write Choice 3 (3) __________________________________________________

Q58 What changes could encourage or increase your participation with peer support?

Click to write Choice 1 (1) __________________________________________________

Click to write Choice 2 (2) __________________________________________________

Click to write Choice 3 (3) __________________________________________________

Q60 Thinking about information needs relating to MS you might have, rate your agreement with the following statement: 'I have received enough information about MS and how to manage MS'

Strongly agree (1)

Agree (2)

Neither agree nor disagree (3)

Disagree (4)

Strongly disagree (5)

Q61 How strongly would you rate your agreement with the following statements

Display This Choice:

If Q1 = I am a caregiver of someone who lives with multiple sclerosis

Display This Choice:

If Q1 = I am a caregiver of someone who lives with multiple sclerosis

Display This Choice:

If Q1 = I live with multiple sclerosis (MS)

Display This Choice:

If Q1 = I live with multiple sclerosis (MS)

|  | Strongly agree (1) | Agree (2) | Neither agree nor disagree (3) | Disagree (4) | Strongly disagree (5) |
| --- | --- | --- | --- | --- | --- |
| Display This Choice:  If Q1 = I am a caregiver of someone who lives with multiple sclerosis  Other caregivers of people with MS are a useful source of information (1) |  |  |  |  |  |
| Display This Choice:  If Q1 = I am a caregiver of someone who lives with multiple sclerosis  Other caregivers of people with MS are a useful source of psychological or emotional support (2) |  |  |  |  |  |
| Display This Choice:  If Q1 = I live with multiple sclerosis (MS)  Other people with MS are a useful source of information (3) |  |  |  |  |  |
| Display This Choice:  If Q1 = I live with multiple sclerosis (MS)  Other people with MS are a useful source of psychological or emotional support (4) |  |  |  |  |  |

Q62 If you would like to, please provide more information on your answers to the above questions?

________________________________________________________________

End of Block: Block 8

Start of Block: Block 9

Q64 Part 5: Your preferences for support

Display This Question:

If Q1 = I live with multiple sclerosis (MS)

Q65 Which of the following peer supports would you be interested in engaging with? (Please check all that apply). I would like to attend or receive:

- Peer support for people with similar symptoms (e.g. progressive neurological illnesses) (1)
- Peer support for people experiencing chronic/ long lasting illness (e.g. chronic illness) (2)
- In-person structured sessions ( e.g. to discuss specific topics about MS with other people with MS) (3)
- In-person informal meetings (e.g. coffee mornings with other people with MS) (4)
- Online structured peer support sessions to discuss specific topics about MS with other people with MS (e.g. conducted on Zoom/ Google Meet/ Microsoft Teams/ Skype) (5)
- Online informal meetings with other people with MS (6)
- Social media private groups (e.g. Facebook MS groups) (7)
- Other social media ( Instagram/ X) (8)
- Messaging support (e.g. WhatsApp/ Telegram/ Emails) (9)
- Other (10)

Display This Question:

If Q1 = I am a caregiver of someone who lives with multiple sclerosis

Q66 Which of the following peer supports would you be interested in engaging with? (Please check all that apply). I would like to attend or receive:

- Peer support for caregivers of people with similar symptoms (e.g. progressive neurological illnesses) (1)
- Peer support for caregivers of people experiencing chronic/ long lasting illness (e.g. chronic illness) (2)
- In-person structured sessions ( e.g. to discuss specific topics about MS with other caregivers of people with MS) (3)
- In-person informal meetings (e.g. coffee mornings with other caregivers of people with MS) (4)
- Online structured peer support sessions to discuss specific topics about MS with other caregivers of people with MS (e.g. conducted on Zoom/ Google Meet/ Microsoft Teams/ Skype) (5)
- Online informal meetings with other caregivers of people with MS (6)
- Social media private groups (e.g. Facebook MS or caregiver groups) (7)
- Other social media ( Instagram/ X) (8)
- Messaging support (e.g. WhatsApp/ Telegram/ Emails) (9)
- Other (10)

Display This Question:

If Q65 = Other

Q67 Please specify, other

________________________________________________________________

Display This Question:

If Q66 = Other

Q68 Please specify, other

________________________________________________________________

Q69 Which of the following peer support groups would you like to attend?

Display This Choice:

If Q1 = I am a caregiver of someone who lives with multiple sclerosis

Display This Choice:

If Q1 = I am a caregiver of someone who lives with multiple sclerosis

Display This Choice:

If Q1 = I am a caregiver of someone who lives with multiple sclerosis

Display This Choice:

If Q1 = I am a caregiver of someone who lives with multiple sclerosis

Display This Choice:

If Q1 = I am a caregiver of someone who lives with multiple sclerosis

Display This Choice:

If Q1 = I live with multiple sclerosis (MS)

Display This Choice:

If Q1 = I live with multiple sclerosis (MS)

Display This Choice:

If Q1 = I live with multiple sclerosis (MS)

Display This Choice:

If Q1 = I live with multiple sclerosis (MS)

Display This Choice:

If Q1 = I live with multiple sclerosis (MS)

|  | Strongly agree (1) | Agree (2) | Neither agree nor disagree (3) | Disagree (4) | Strongly disagree (5) |
| --- | --- | --- | --- | --- | --- |
| Display This Choice:  If Q1 = I am a caregiver of someone who lives with multiple sclerosis  People caring for people with the same MS type as my care recipient (1) |  |  |  |  |  |
| Display This Choice:  If Q1 = I am a caregiver of someone who lives with multiple sclerosis  People caring for people with a similar level of disability with my care recipient (2) |  |  |  |  |  |
| Display This Choice:  If Q1 = I am a caregiver of someone who lives with multiple sclerosis  People with similar ages to me (3) |  |  |  |  |  |
| Display This Choice:  If Q1 = I am a caregiver of someone who lives with multiple sclerosis  People in similar life stages or circumstances to me (e.g. students, working parents of young children, retired) (4) |  |  |  |  |  |
| Display This Choice:  If Q1 = I am a caregiver of someone who lives with multiple sclerosis  People living in in the same locality as me (5) |  |  |  |  |  |
| Display This Choice:  If Q1 = I live with multiple sclerosis (MS)  People with the same MS type to me (6) |  |  |  |  |  |
| Display This Choice:  If Q1 = I live with multiple sclerosis (MS)  People with MS having a similar level of disability with me (7) |  |  |  |  |  |
| Display This Choice:  If Q1 = I live with multiple sclerosis (MS)  People with MS with similar ages to me (8) |  |  |  |  |  |
| Display This Choice:  If Q1 = I live with multiple sclerosis (MS)  People with MS in similar life stages or circumstances to me (e.g. students, working parents of young children, retired) (9) |  |  |  |  |  |
| Display This Choice:  If Q1 = I live with multiple sclerosis (MS)  People with MS living in the same locality as me (10) |  |  |  |  |  |

Q70 If attending an online support group, would you prefer to remain anonymous?

No (1)

Unsure (2)

Yes (3)

Q71 How many people do you think would be best to include in a peer support group

________________________________________________________________

Q72 Please rate your agreement with the following statement: ' I am confident using digital devices and the internet'

Strongly agree (1)

Agree (2)

Neither agree nor disagree (3)

Disagree (4)

Strongly disagree (5)

Q73 Finally, if there is anything else you would like to add in relation to any of the questions in this survey, please do so below

________________________________________________________________

**(Supplementary material) Table S2** Correlation matrix, associations of UCLA-3, OSLO-3, WHO-5, and in-person and online peer support (PS) engagement

|  | **In-person PS engagement** | **Online PS engagement** | **WHO-5** | **UCLA-3** | **OSLO-3** |
| --- | --- | --- | --- | --- | --- |
| **In-person PS engagement** | 1.000 |  |  |  |  |
| **Online PS engagement** | 0.489*** | 1.000 |  |  |  |
| **WHO-5** | 0.197** | 0.100 | 1.000 |  |  |
| **UCLA-3** | -0.207** | 0.053 | -0.491*** | 1.000 |  |
| **OSLO-3** | 0.170* | 0.181* | 0.492*** | -0.501*** | 1.000 |

*p* = ***= 0.000, **= 0.005, *= 0.01
